# Draft Genome Sequences of Nonclinical and Clinical *Enterobacter cloacae* Isolates Exhibiting Multiple Antibiotic Resistance and Virulence Factors

**DOI:** 10.1128/genomeA.01218-17

**Published:** 2017-11-09

**Authors:** Mitali Mishra, Shashank Patole, Harapriya Mohapatra

**Affiliations:** School of the Biological Sciences, National Institute of Science Education and Research, HBNI, Bhubaneswar, Odisha, India

## Abstract

*Enterobacter* spp. have been implicated as opportunistic pathogens which over the years have gained resistance toward most of the available therapeutic drugs. We sequenced two multidrug-resistant *Enterobacter cloacae* isolates harboring multiple efflux pump genes. These isolates exhibited strain-specific modulation of efflux pump protein expression.

## GENOME ANNOUNCEMENT

*Enterobacter* spp. are ubiquitously present in the environment and are important nosocomial pathogens primarily affecting immunocompromised patients (1). Their ability to evade the biocidal action of several groups of antibiotics has resulted in their inclusion in the ESKAPE (*Enterococcus faecium*, *Staphylococcus aureus*, *Klebsiella pneumoniae*, *Acinetobacter baumannii*, *Pseudomonas aeruginosa*, and *Enterobacter* species) group of pathogens (2). *Enterobacter* spp. are implicated as etiological agents in a plethora of infections, such as urinary tract infections, meningitis, septicemia, bacteremia, wound infection, lower gastrointestinal infections, and nosocomial infections (3). Previously, we studied the association between multiple-antibiotic resistance and virulence in environmental Gram-negative bacterial isolates, including those belonging to *Enterobacter* species (4). From the aforementioned study, we selected the environmental *Enterobacter cloacae* isolate DL4.3 (GenBank accession number JQ912514) that showed a multidrug resistance phenotype. *Enterobacter cloacae* EspIMS6 was a urinary tract infection isolate received from a tertiary-care hospital in Bhubaneswar, Odisha, India, that exhibited extreme drug resistance. A decrease in MIC values for antibiotics in the presence of the proton motive gradient disrupter carbonyl cyanide *m*-chlorophenylhydrazone indicated a possible involvement of efflux pumps in mediating resistance in both the isolates. Immunoblotting has shown strain-specific modulation of AcrAB-TolC efflux proteins in response to pH and cephalosporin antibiotics. This study reports the whole-genome sequences of the above-mentioned two isolates, which is a prerequisite for understanding the molecular basis of antibiotic resistance, the repertoire of efflux genes harbored by the organisms, the efflux pump regulatory proteins encoded by the organisms, and the contribution of efflux proteins in physiological functions in the cell, particularly in the context of the source of isolation.

Genomic DNA from both isolates was extracted using the Gentra Puregene Yeast/Bact. kit (Qiagen GmbH), according to the manufacturer’s instructions. Whole-genome sequencing was carried out at the laboratory of Thermo Fisher Scientific India, Gurgaon, India. Briefly, libraries for individual genomes were prepared using the workflow prescribed by the Ion Xpress Plus fragment library kit (Thermo Fisher Scientific, USA). Subsequently, the reads were amplified using the Ion OneTouch 2 system (Thermo Fisher Scientific) and sequenced using the Ion S5 system (Thermo Fisher Scientific). A total of 822,417,719 and 859,537,668 bases were obtained in the form of 2,444,256 and 2,549,556 reads, with average read lengths of 336 and 337 bp for EspIMS6 and DL4.3, respectively. The reads were assembled using the SPAdes algorithm version 3.1.0 (4) into 203 and 145 contigs, with average sizes of 148,694 and 122,602 bases for EspIMS6 and DL4.3, respectively.

The genomes were uploaded to the Rapid Annotations using Subsystems Technology (RAST) server (5, 6) that was used to annotate the genomes, which were 5,296,869 and 4,820,048 bp in size, with 54.7% and 54.9% G+C content for EspIMS6 and DL4.3, respectively. EspIMS6 and DL4.3 were found to have 5,013 and 4,545 protein-coding genes, of which 4,006 and 3,731 were assigned functions, respectively. Further, 102 and 104 genes were found to code for RNA in EspIMS6 and DL4.3, respectively. Additionally, the sequences have been submitted to the Prokaryotic Genome Annotation Pipeline (PGAP) (7). The availability of genome sequences would enable us to undertake genomic comparisons of *Enterobacter cloacae* isolates from nonclinical and clinical sources.

### Accession number(s).

This whole-genome shotgun project has been deposited in DDBJ/EMBL/GenBank under the accession numbers NMPX00000000 and NRIS00000000 for *Enterobacter cloacae* isolates DL4.3 and EspIMS6, respectively. The versions presented here are the first draft versions for both the genomes and are publicly available.
